# Theoretical Modeling of the Structure Formation in Adsorbed Overlayers Comprising Molecular Building Blocks with Different Symmetries

**DOI:** 10.3390/molecules30040866

**Published:** 2025-02-13

**Authors:** Paweł Szabelski

**Affiliations:** Department of Theoretical Chemistry, Institute of Chemical Sciences, Faculty of Chemistry, Maria Curie-Skłodowska University in Lublin, Pl. M.C. Skłodowskiej 3, 20-031 Lublin, Poland; pawel.szabelski@mail.umcs.pl; Tel.: +48-081-537-77-62; Fax: +48-081-537-56-85

**Keywords:** adsorption, self-assembly, functional molecules, binary mixtures, Monte Carlo simulations

## Abstract

Controlling the geometry and functionality of multi-component self-assembled superstructures on surfaces is a complex task that requires numerous experimental tests. In this contribution, we demonstrate how computer modeling can be utilized to preselect functional tectons capable of forming low-dimensional architectures with tailored features. To this end, coarse-grained Monte Carlo simulations were conducted for a mixture of tripod and tetrapod units, each equipped with discrete centers for short-range directional interactions, and adsorbed onto a (111) crystalline substrate. The calculations conducted for various isomers of the tetrapod molecule revealed qualitatively distinct self-assembly scenarios, including mixing and segregation, depending on the directionality of interactions assigned to this tecton. The resulting superstructures were classified, and their formation was monitored using temperature-dependent metrics, such as coordination functions. The findings of this study contribute to a better understanding of the on-surface self-assembly of molecules with differing symmetries and can aid in the design of bicomponent overlayers for specific applications.

## 1. Introduction

Precisely controlling the positioning of molecular building blocks in adsorbed overlayers is a challenging task, crucial for the fabrication of low-dimensional functional nanostructures. One approach to achieving this goal is the on-surface self-assembly of organic molecules equipped with well-defined interaction centers. It has been demonstrated in numerous cases that fine-tuning molecular features—such as the shape, size, number, and intramolecular distribution of functional groups—combined with the adjustment of external parameters like temperature, solvent type, and surface coverage enables the fabrication of highly ordered 2D assemblies with a wide variety of structural motifs. A good example of this is planar periodic porous networks with nano-cavities, whose shape and size can be tuned by selecting an appropriate molecular tecton [1,2]. In porous networks, the contributing molecules are arranged periodically, while also creating an array of empty sites that can be filled with foreign matter possessing predefined properties, such as optical or magnetic characteristics [3,4].

In general, two main strategies have been employed to create mixed guest–host systems on solid substrates. A more straightforward approach is to create a porous network using a single tecton, then adsorb guest molecules that fit within the pores [5,6]. A key advantage of this method is that the (exact or approximate) arrangement of guest molecules is known in advance, as it is dictated by the architecture of the porous network (except in the less frequent but possible scenario where the guest molecules deform the network, etc.). The aforementioned sequential creation of mixed molecular overlayers has been successfully demonstrated in numerous adsorbed systems, both in organic liquid phases and under ultra-high vacuum conditions [3].

The second approach to creating multicomponent arrays on surfaces is much more challenging, as it involves the simultaneous adsorption and self-assembly of multiple components, the outcome of which is often difficult to predict in advance [7,8,9]. Specifically, the morphology of the final assembly is usually highly sensitive to the composition of the adsorbing mixture (mole fractions of the components), surface coverage, and temperature. As a result, even small modifications to these parameters can cause drastic changes in the architecture of the obtained superstructures, leading to complex phase coexistence within these systems [10]. This applies, for example, to the mixing and demixing of components, as well as polymorphism, which can occur under specific external conditions [7]. Despite these difficulties, experimental evidence shows that careful tuning of the proportions of the contributing tectons can effectively create molecular arrays with varying structures on graphite and metallic surfaces. For example, to that end, star-shaped network-forming molecules have often been adsorbed alongside pore-filling species such as coronene or graphene [3,11]. Moreover, recent studies have also examined multicomponent mixtures comprising molecules of three or four types [9,12]. In these systems, the additional species formed complex hierarchical networks that facilitated the occlusion of guest molecules (e.g., coronene). Multicomponent systems have also been used to investigate the effects of chirality and achirality of the building blocks, as well as chirality transfer to the growing mixed superstructures [13,14]. In the above examples, the mixtures, in addition to the main network-forming component, included molecules (modifiers) of varying shapes and sizes, which regulated the distance between occluded guest species [15].

Experimental studies aimed at creating multicomponent superstructures with predefined properties on surfaces require careful selection of molecular tectons and precise programming of the interactions between them. In practice, assembling a new adsorbed superstructure with assumed properties often relies on a trial-and-error approach, where a series of probe building blocks are tested. This procedure can be tedious, as each candidate molecule must be synthesized and its self-assembly examined using scanning probe microscopy. Even for single-component systems, where a functional molecule can have several positional isomers, testing each one experimentally can be challenging [16]. Clearly, the situation becomes much more complex when two or more components can exist in various isomeric forms.

A useful approach for predicting possible outcomes of self-assembly in adsorbed overlayers has been the use of theoretical methods, particularly computer simulations. Computational methods, such as molecular dynamics [17,18,19,20] and Monte Carlo simulations [7,15,21,22,23,24,25,26,27], have proven to be especially helpful in modeling large molecular assemblies comprising various building blocks. These methods allow for the easy modification of parameters that describe key properties of interacting molecules, including their structural characteristics (shape and size) and functionality (number and location of interaction centers). As a result, the initial selection of candidate tectons capable of forming a superstructure with a predefined morphology can be greatly facilitated. This applies particularly to simplified coarse-grained models, which enable the rapid testing of extensive sets of probe molecules.

To date, coarse-grained Monte Carlo models have been successfully used to reproduce 2D self-assembled structures (also multicomponent) driven by interactions such as hydrogen bonding [22,26], metal–organic ligand coordination [24,25], halogen bonding [27], and van der Waals forces [7,15,21]. In these calculations, surprisingly complex ordered patterns have been observed, ranging from porous networks and hierarchical nested structures to even fractal aggregates [28]. It has also been observed that slight modifications in the interaction pattern—determined by the directionality of interactions resulting from the specific positions of functional groups in a tecton—can lead to substantial changes in the morphology of the corresponding assembly. In this context, studying the effect of programmable interaction directions provided by active molecular centers is essential for understanding and predicting pattern formation in related one- and multicomponent systems adsorbed on solid surfaces.

In this contribution, we focus on the on-surface self-assembly of a binary mixture consisting of molecular building blocks that differ significantly in symmetry. Specifically, we consider a mixture of tripod-shaped *C*_3v_-symmetric tectons with terminal active centers that have fixed interaction directions and an analogous tetrapod unit with a D_2h_-symmetric backbone and adjustable interaction directions. Our main objective is to examine how different assignments of interaction directions in the tetrapod tecton, corresponding to various positional isomers, affect the structure formation in the mixed overlayer. In particular, we identify which of these assignments lead to effects such as phase separation, network formation, and molecular occlusion. The results reported herein can be useful for custom designing 2D molecular binary superstructures stabilized by short-range intermolecular interactions. These findings can facilitate the preliminary selection of organic building blocks capable of forming overlayers with desired structural properties.

## 2. Results and Discussion

The various ways in which the four interaction directions can be encoded in the tetrapod molecule (with three possibilities for each of the four arms of **B**) theoretically result in 81 positional isomers. Due to the mirror symmetry of certain pairs of these planar structures, the total number of distinct isomers of **B** is significantly reduced, resulting in 27 distinct tectons. Figure S1 of the Supporting Information section presents these tectons along with their corresponding number codes. To explore possible structures formed by coadsorbed **A** and **B**, we conducted simulations for all 27 isomers of the latter component. Our primary goal was to identify similarities in the behavior of certain groups of isomers of **B** and to characterize and classify the resulting superstructures. We also attempted to connect the intrinsic molecular properties of **B** (i.e., the sequence of interaction directions) with its ability to either segregate or form mixed assemblies with **A**.

### 2.1. Formation of Mixed Structures

**Figure 1:**
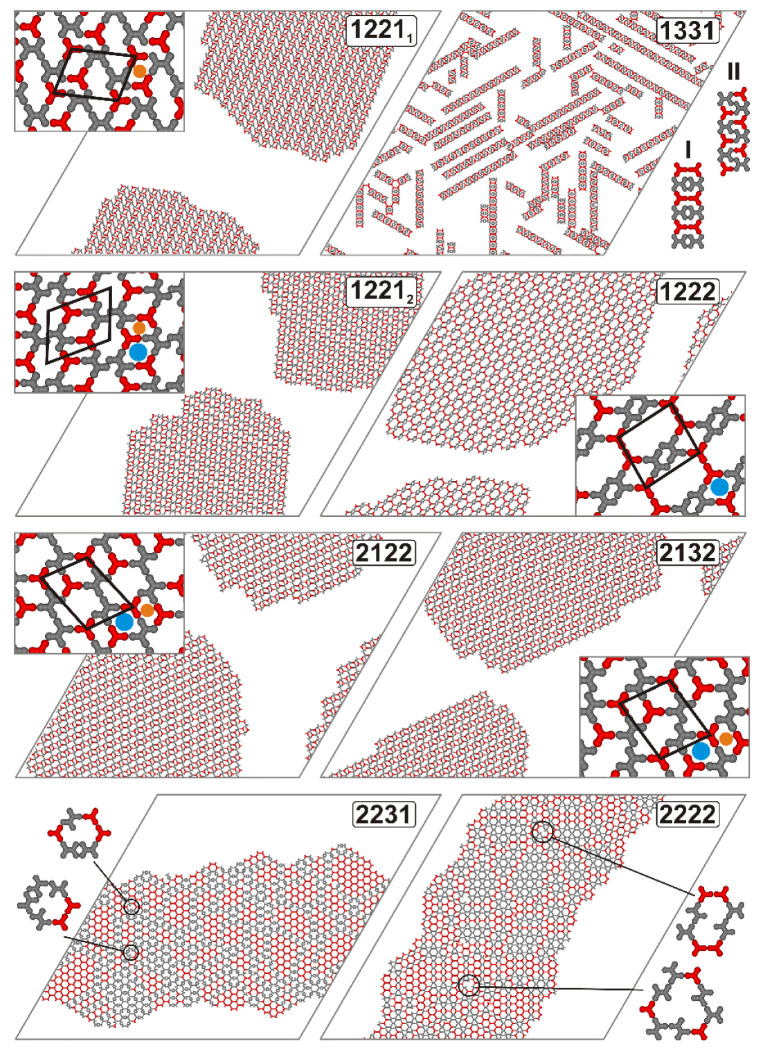
Snapshots of the adsorbed overlayers consisting of 800 molecules of **A** (red) and 800 molecules of **B** (gray), with the latter component exhibiting different sets of interaction directions, as indicated in the respective panels. The thick black lines outline the unit cells of the corresponding ordered networks. Polymorphic networks of **1222** are designated with subscripts 1 and 2. The blue and orange dots represent the types of pores that are shared among different networks. Two possible ladder structures of **1331** are designated as **I** and **II**. The insets in the bottom panels show examples of defective pores formed in the systems comprising **2231** and **2222**. L=200, T=0.01, θ=0.20.

A distinct kind of phase behavior for the coadsorbed components **A** and **B** is the formation of ordered arrays with well-defined periodicity. This type of ordering results in a regular spatial arrangement of **A** and **B**, which can be advantageous, for instance, in creating reproducible 2D connections with emergent properties. Our simulations revealed that out of the 27 possible isomers of **B**, only three were capable of exclusively forming extended mixed networks as dominant superstructures, that is, **1221**, **1222**, **2122**, and **2132**. Figure 1 illustrates these networks, formed in an equimolar mixture of **A** and **B**, consisting of a total of 1600 molecules. Isomers **1222**, **2122**, and **2132** self-assembled into single mixed networks, while for unit **1221**, two polymorphic structures, **12**,**21_1_** and **1221_2_**, were observed in the simulations. The latter polymorph was formed significantly less frequently, occurring in only 16 out of 50 system replicas. Networks **1221_1_**, **1221_2_**, **1222**, **2122**, and **2132** share similar properties, including a parallelogram shape and a 2**A** + 2**B** composition of the unit cell. Specifically, the dimensions of the unit cells are $\sqrt{31}\times \sqrt{67}$, ∝=63.27°; $\sqrt{37}\times 7$, ∝=63.50°; $\sqrt{61}\times 7$, ∝=86.33°;$\;\sqrt{37}\times \sqrt{67}$, ∝=73.07°; and $\sqrt{37}\times \sqrt{57}$, ∝=78.70°, respectively. Given the same composition of each unit cell, the differences in the density of the corresponding porous phases are influenced by the area of these cells so that $\rho_{1221_{1}}=0.491$, $\rho_{1221_{2}}=0.525$, $\rho_{1222}=0.366$, $\rho_{2122}=0.420$ and $\rho_{2132}=0.444$. Another common feature of the networks created by **1221**, **1222**, **2122**, and **2132** is that they are heteroporous, consisting of three types of pores each. These void spaces are roughly deformed parallelograms (see the insets in Figure 1), with rims defined by either 2**A** + 2**B** or 2**A** + 4**B** molecules. Interestingly, some of the pores are common to certain networks shown in Figure 1. For example, the small 2**A** + 2**B** pores marked with orange dots are present in all the networks except for **1222**, while the similar 2**A** + 2**B** pores indicated by blue dots are common to **1221_2_**, **1222**, **2122,** and **2132**. Among these networks, the one formed by **1222** features the largest pores, encompassing 32 lattice sites.

The observed tendencies for pore formation can be explained based on the analysis of the interaction directions encoded in the considered tectons. To potentially create the medium-sized pores marked in blue, a **B** isomer must have two directional interactions **2** on either side (or both sides) of the linear core of this tecton. This implies that in the code *abcd* either b=c=2 or a=d=2. Indeed, this condition is satisfied for **1222**, **2122**, and **2132**, all of which form the aforementioned pores. Moreover, unit **1221** also meets the structural criterion described above and has been shown to form medium-sized cavities in the less abundant network polymorph denoted with subscript 2. Similarly, to create the small pores marked in orange, **B** has to be equipped with specific interaction directions assigned to a pair of arm segments connected to one end of the linear backbone. In this case, one direction should correspond to the *para* position, while the other should correspond to the *meta* position parallel to the core. This requirement can be met in several ways: a=2 and b=1; a=3 and b=2; c=2 and d=1; and c=3 and d=2. Note that all of the **B** tectons satisfy one or more of these conditions, such as in the cases of **1221** and **2122**, or in the case of **2132** (corresponding directions are underlined).

The specific sets of interaction directions in units **1221**, **1222**, **2122**, and **2132** enabled the corresponding networks to propagate in the three principal directions of the lattice without defect formation. On the other hand, for the next two isomers, **2222** and **2231**, the simulations produced extended mixed networks that lacked long-range order and contained numerous defects. The two bottom panels of Figure 1 illustrate these molecular assemblies. In the case of **2231**, a prominent structural feature of the corresponding network is the presence of local domains of **A**, surrounded by double rows of **B**, which glue and stabilize the entire architecture. However, numerous vacancy defects can be found there, with examples shown in the insets of the respective panels. These imperfections are primarily associated with the incomplete coordination of the tetrapod molecules (typically one or two; see the inset), which expose their dangling arms within the structural feature of the corresponding network in the presence of local domains of **A**, surrounded by double rows of **B**, which glue and stabilize the entire architecture. However, numerous vacancy defects can be found there, with examples shown in the insets of the respective panels. These imperfections are primarily associated with the incomplete coordination of the tetrapod molecules (typically one or two; see the inset), which expose their dangling arms within the pores. A somewhat different outcome of mixing was observed for tecton **2222**, where the occlusion of **A** domains was practically absent, and wide interconnecting rows of **B** (and potentially **A**) were not formed. As a result, components **A** and **2222** were more widely dispersed within the network, leading to the observation of various defects, including the two largest shown in the pores. A somewhat different outcome of mixing was observed for tecton **2222**, where the occlusion of **A** domains was practically absent, and wide interconnecting rows of **B** (and potentially **A**) were not formed. As a result, components **A** and **2222** were more widely dispersed within the network, leading to the observation of various defects, including the two largest shown in the inset next to the bottom right panel.

Regarding the relationship between the interaction directions of **2231** and **2222** and the morphology of the corresponding assemblies, some connections can be established. In the case of the first tecton, the formation of double molecular rows joining **A** domains is associated with the presence of two parallel interaction directions assigned to segments *d* and *c* of this tetrapod unit. This pair of parallel interaction directions (**31**) excludes the possibility of attaching two **A** molecules simultaneously. As a result, bimolecular complexes of **2231** are predominantly formed, and this basic structural motif is either incorporated into the rows or contributes to small fragments of a Kagome-like network of pure **B**, visible in the left part of the corresponding snapshot. For **2222**, the significant structural complexity of the obtained network can be paradoxically associated with the high symmetry of this molecule. In this case, the centrosymmetric properties of **2222**, with each interaction direction pointing along the arm, allow for a vast range of **A**–**B** configurations, leading to a multiverse of regular and deformed pores with undercoordinated molecules.

The final example of mixed structures created during all the simulation runs is the ladders comprising isomer **1331**. Interestingly, we observed two polymorphic forms of these structures, **I** and **II**, differing in the relative arrangements of components **A** and **B**, shown in the respective inset. In one type of these ladders (**I**), pairs of molecules of **A** and pairs of molecules of **1331** were stacked alternately, parallel to each other and oriented perpendicular to the ladder growth direction. In the second type of ladders (**II**), the alternating pairs of tectons were tilted, and, furthermore, there was no direct contact between the molecules of **A**. The inability of isomer **1331** to co-assemble into extended networks primarily stems from the presence of interaction directions **1** and **3**, which are associated with segments *a* and *b*. This pair of interaction directions gives isomer **1331** an anchor-like shape, and due to steric exclusion, when the ladder is formed, it allows for the attachment of molecules of **A** but not **B**. If two molecules of **A** are linked to **1331** in this manner, the structure can only grow in one direction, as the edge of the ladder becomes inactive with no exposed active segments.

**Figure 2:**
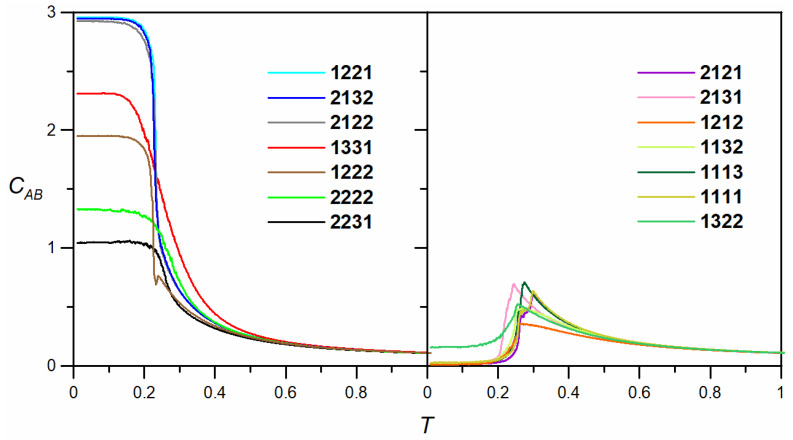
The effect of temperature on the average number of interactions formed by a single tripod molecule of **A** with neighboring molecules of **B**, *C**_AB_***. The curves in the left and right panels correspond to the isomers of **B**, where mixing (Figure 1) and separation were observed, respectively. L=200, T=0.01, θ=0.20.

The characteristic structural features of the self-assembled mixtures mentioned above are captured in the corresponding quantitative descriptors, one of which is the average number of molecule **B** units coordinating with each molecule of **A**, *C**_AB_***. This parameter approaches zero when separation occurs between **A** and **B**, and it is close to 3 when the components are perfectly mixed. Figure 2 illustrates the changes in *C**_AB_*** caused by decreasing temperature, as observed for the seven isomers shown in Figure 1.

For the three tectons **1221**, **2122**, and **2132**, the curves nearly overlap, indicating the formation of similar heteroporous periodic networks (see Figure 1). The corresponding curves rise sharply as the temperature approaches approximately 0.23 and level off at a plateau close to 3. The sharp increase in *C**_AB_*** signifies the condensation of the adsorbed components and the highly efficient incorporation of these units into the growing ordered domain. The contact value of *C**_AB_*** (i.e., as *T* → 0), which is approximately 3, confirms the previous common observation for the mixed networks containing **1221**, **2122**, and **2132**, where each molecule of **A** interacts with three neighboring molecules of **B**. The slightly lower position of the plateau, just below 3, arises from the finite size of the porous domains, which include peripheral undercoordinated molecules of **A**. For isomer **1222**, the plateau of the coordination curve is significantly lowered to approximately 2, indicating that in the corresponding network, each molecule of **A** coordinates with two molecules of **B**.

The formation of ladders consisting of isomer **1331** resulted in a coordination curve that was less steep and reached a plateau lower than those of the network-forming tectons **1221**, **2122**, and **2132** (~3) but higher than that of **1222** (~2). In this case, the self-assembly of ladders occurred at somewhat higher temperatures, due to the increased stability of these emerging structures (for the ladders, disintegration can only begin at the ends, making it less efficient compared to the disintegration of network nuclei). The corresponding contact value of *C**_AB_***, approximately 2.31, results from the different coordination patterns of **A** in the two types of ladders shown in the inset. In the case of ladders of type **I**, a molecule of **A** is in contact with two molecules of **B**, while for ladders of type **II**, it is fully coordinated by **B**. From an energetic perspective, the two types of ladders are identical, as each structure has every contributing molecule fully coordinated (except for the terminal ones). Consequently, the occurrence of ladders of type **I** and **II** should be equally probable, leading to a theoretical contact value of *C**_AB_*** = 2.5. The observed value of 2.31 is close to the theoretical prediction, and the difference arises mainly from the significant dispersion of ladders, which contain several undercoordinated molecules of **A** at the ends.

The irregular defective networks formed by **2222** and **2231** exhibit temperature dependencies that predict much lower heterogeneous coordination compared to the isomers discussed so far. Specifically, the obtained contact values of *C**_AB_*** are equal to 1.32 and 1.05, respectively. These low values are a direct consequence of the clustering of components **A** and **B** in the networks depicted in Figure 1. This effect results in the presence of numerous units of **A** that have no interaction with **B**—within the occluded **A** fragments—and undercoordinated molecules of **A** at the boundaries of these fragments, typically interacting with two units of **B**. Thus, for both **2222** and **2231**, the net average coordination of **A** is close to 1, with the latter molecule exhibiting a stronger tendency to form occlusions; *C**_AB_*** = 1.05. The shape of the corresponding curves shows that the *C**_AB_***(*T*) calculated for **2231** is steeper, which may be attributed to the more efficient formation (condensation) of occluded **A** domains. For **2222**, the analogous curve indicates that the linkage of **A** begins at higher temperatures, which can be attributed to the increased ease of forming mixed pores by the centrosymmetric **2222** unit. However, as previously mentioned, although the **2222** isomer can be more readily incorporated into the growing network and better mixed with **A**, the resulting assembly remains highly disordered.

### 2.2. Segregation of Adsorbed Components

**Figure 3:**
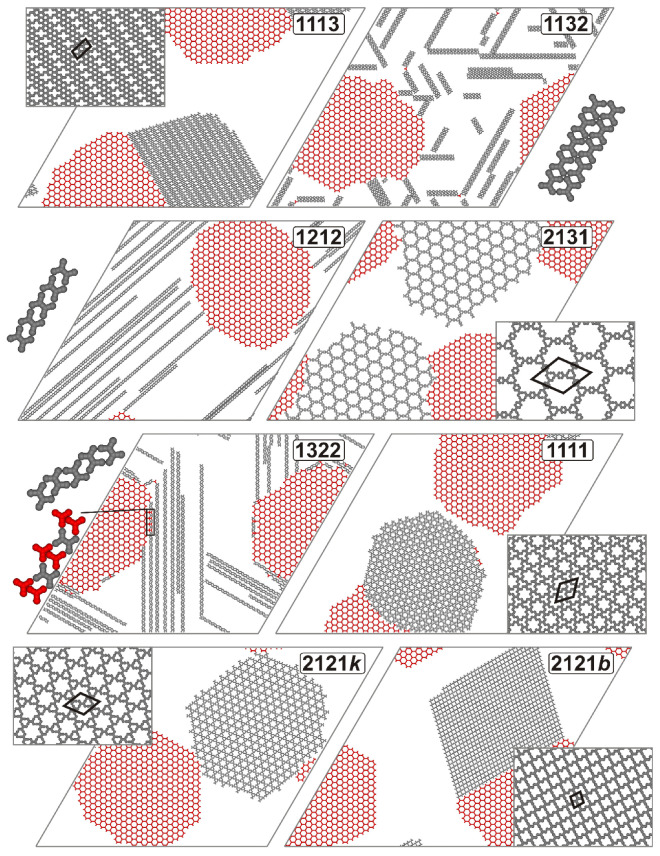
Snapshots of the adsorbed overlayers consisting of 800 molecules of **A** (red) and 800 molecules of **B** (gray), with the latter component exhibiting different sets of interaction directions, as indicated in the respective panels. The thick black lines outline the unit cells of the corresponding ordered networks. Magnified fragments of the chains and ladders formed by **1212**, **1132,** and **1322** are displayed next to the corresponding snapshots. For the **2121** unit, two network polymorphs—Kagome (***k***) and brick wall (***b***)—observed in the simulations are presented in the bottom panels. L=200, T=0.01, θ=0.20.

In this section, we discuss the qualitatively distinct self-assembly behavior of the adsorbed units **A** and **B**, which leads to their segregation. Specifically, we analyze representative examples of systems where various types of coexisting structures, composed solely of either **A** or **B**, were observed. The first type of phase behavior predicted for the given set of external parameters is the formation of a pure hexagonal network of **A [23]**, alongside a separate domain of single-type **B**. Figure 3 illustrates this effect for isomer **1113**, which forms a densely packed brick-wall structure with a parallelogram unit cell of dimensions $3\times \sqrt{57}$ and an angle of ∝=53.41°. In the snapshot presented, additional, though very limited, stabilization of the domains is achieved through their direct contact. At the straight interphase boundary, molecules of **A** and **B** establish sparse interactions, with the latter primarily utilizing direction 1, which is assigned to segment *b*. Due to the distinct periodicities of the contacting domains, these interactions occur between every second molecule of **A** and every fourth molecule of **B**, resulting in only a small contribution to the overall potential energy. The aforementioned segregation scenario was also observed for isomers **1213** and **1313**, which formed structurally similar although more closely packed domains (see Figure S2 of the Supporting Information section).

In contrast to the relatively dense phases formed by **1113**, **1213**, and **1313**, the self-assembly of isomer **2131** resulted in a segregated hexagonal network with exceptionally large, regular pores, as shown in Figure 3. This network features a large rhombic unit cell (∝=60.00°) with a side length of $\sqrt{148}$, accommodating six molecules. A single hexagonal pore in the **2131** network encompasses 91 empty lattice sites, which is significantly larger compared to the hexagonal network of **A**, where each pore spans only 19 lattice sites. Moreover, the pores formed by **2131** were identified as the largest among all the void spaces observed in this study. A periodic hexagonal network resembling that of **2131** was also formed by tecton **1112**, featuring large pores spanning 67 lattice sites (see Figure S2). A similar porous structure, albeit with smaller hexagonal void spaces consisting of 13 lattice sites, was observed for isomer **1111**. In this case, the resulting network exhibited a Kagome topology and was characterized by a $\sqrt{39}\times \sqrt{39}$ unit cell with an angle of ∝=60.00°, accommodating three molecules, as shown in Figure 3.

A more complex segregation pattern of molecules **A** and **B** was associated with the coexistence of the pure **A** network and one of the two possible polymorphs of **B**-based networks. An example of the outcome of this segregation process is illustrated in the two bottom panels of Figure 3, which depict isomer **2121**. In this case, the molecules of **B** formed either the Kagome (*k*) network, characterized by large hexagonal pores, or the brick-wall network (*b*), which features smaller parallelogram pores. The latter superstructure was less prevalent, appearing in 11 out of 50 system replicas. Networks **2121*k*** and **2121*b*** were characterized by unit cells of dimensions 7 by 7 (∝=60.00°) and $\sqrt{12}$ by $\sqrt{13}$ (∝=76.10°), respectively. The brick-wall network **2121*b*** was frequently observed in contact with the porous domain of **A**, as illustrated in the corresponding panel. For this type of network, similar to **1113**, there was the possibility of forming a straight interface region where every second molecule of **A** interacted with every fourth molecule of **B** (utilizing direction **2** at segment *c*). Although isomer **2121** exhibited a tendency to form more diverse structures, we never observed it mixing with **A** during the simulations. The centrosymmetric molecules of **2121** were unable to intermix with **A**, primarily due to the presence of interaction directions that were not collinear with their arms. These off-arm interactions hindered the extended growth of mixed networks, as the tripod molecules were unable to coordinate effectively with **B** and form closed pores without vacancy defects. A similar segregation scenario, resulting in two polymorphic porous networks, was observed in the case of the **1121** isomer, as illustrated in Figure S2. However, in this case, the alternative pure networks shared the same six-fold symmetry but differed in pore size.

In contrast to the relatively dense phases formed by **1113**, **1213**, and **1313**, the self-assembly of isomer **2131** resulted in a segregated hexagonal network with exceptionally large, regular pores, as shown in Figure 3. This network features a large rhombic unit cell (∝=60.00°) with a side length of $\sqrt{148}$, accommodating six molecules. A single hexagonal pore in the **2131** network encompasses 91 empty lattice sites, which is significantly larger compared to the hexagonal network of **A**, where each pore spans only 19 lattice sites. Moreover, the pores formed by **2131** were identified as the largest among all the void spaces observed in this study. A periodic hexagonal network resembling that of **2131** was also formed by tecton **1112**, featuring large pores spanning 67 lattice sites (see Figure S2). A similar porous structure, albeit with smaller hexagonal void spaces consisting of 13 lattice sites, was observed for isomer **1111**. In this case, the resulting network exhibited a Kagome topology and was characterized by a $\sqrt{39}\times \sqrt{39}$ unit cell with an angle of ∝=60.00°, accommodating three molecules, as shown in Figure 3.

A more complex segregation pattern of molecules **A** and **B** was associated with the coexistence of the pure **A** network and one of the two possible polymorphs of **B**-based networks. An example of the outcome of this segregation process is illustrated in the two bottom panels of Figure 3, which depict isomer **2121**. In this case, the molecules of **B** formed either the Kagome (*k*) network, characterized by large hexagonal pores, or the brick-wall network (*b*), which features smaller parallelogram pores. The latter superstructure was less prevalent, appearing in 11 out of 50 system replicas. Networks **2121*k*** and **2121*b*** were characterized by unit cells with dimensions of 7 by 7 (∝=60.00°) and $\sqrt{12}$ by $\sqrt{13}$ (∝=76.10°), respectively. The brick-wall network **2121*b*** was frequently observed in contact with the porous domain of **A**, as illustrated in the corresponding panel. For this type of network, similarly to **1113**, there was the possibility of forming a straight interface region where every second molecule of **A** interacted with every fourth molecule of **B** (utilizing direction **2** at segment *c*). Although isomer **2121** exhibited a tendency to form more diverse structures, we never observed it mixing with **A** during the simulations. The centrosymmetric molecules of **2121** were unable to intermix with **A**, primarily due to the presence of interaction directions that were not collinear with their arms. These off-arm interactions hindered the extended growth of mixed networks, as the tripod molecules were unable to coordinate effectively with **B** and form closed pores without vacancy defects. A similar segregation scenario, resulting in two polymorphic porous networks, was observed in the case of the **1121** isomer, as illustrated in Figure S2. However, in this case, the alternative pure networks shared the same six-fold symmetry but differed in pore size.

The last type of segregation refers to the formation of the **B**-based structures with lower dimensions, such as chains and ladders. The growth of simple straight chains was observed for isomers **1212** and **1322,** as illustrated in Figure 3. This type of self-assembly of **B** was also evident for tecton **3131**, as shown in Figure S2. In this context, it is worth noting that the self-assembly of chains was achievable for tectons where pairs of parallel interaction directions were positioned on opposite sides of tecton **B**, either vertically or horizontally. Among the **27** isomers of B, only three possess this property: the centrosymmetric units **1212** and **3131**, as well as isomer **1322**, which exhibits a plane of symmetry. The complementary interaction directions assigned to pairs of segments located on opposite sides of the molecular core (**1212**, **1322**) or to segments connected to different parts of the core (**3131**) dictate the directional growth of the corresponding chains. This type of linkage excludes the possibility of any bending or branching, resulting in straight chains that can grow along the three principal directions of the lattice.

Regarding ladder formation, such structures were observed in the case of isomer **1132** (Figure 3), as well as for tectons **1231** and **1133** (c.f. Figure S2). Depending on the assigned interaction pattern, the molecules within the ladders were either oriented along the propagation direction (**1132**, **1133**) or tilted by 60 degrees relative to it (**1231**). Similarly to the chain-forming isomers, the self-assembly of ladders consistently produced straight structures, with the ends serving as the only growth points.

To understand the origin of the distinct segregation scenarios discussed above, let us take a closer look at the sets of interaction directions assigned to the corresponding tectons. In general, the formation of separate **B**-structures by certain isomers is primarily attributed to their inability to be fully or partially coordinated by **A** molecules, preventing the development of a unified molecular architecture. This situation is evident for **B**-tectons whose interaction directions are entirely incompatible with those of **A** in this regard. As mentioned earlier, the most straightforward example involves the chain-forming isomers **1212**, **1322**, and **3131**, which are characterized by pairs of parallel interaction directions. For the remaining types of segregated **B**-structures, such general conclusions are much more difficult to draw and should be drawn with caution. For example, in the case of ladders, a potentially relevant feature of a tecton is the presence of parallel, oppositely oriented interaction directions on one side of the core, as takes place for **1132** and **1133**. In these tectons, segments *b* and *c* are assigned the interaction directions **1** and **3**, which align with the aforementioned criterion. The interaction between segments *b* and *c* of neighboring molecules facilitates the formation of a linear edge of the ladder, thereby stabilizing one strand. To connect a pair of strands, the tecton must be equipped with a complementary pair of interaction directions, allowing for the formation of arm-based rungs. For molecules **1132** and **1133**, this is achieved through the pair of parallel and crossing interaction directions assigned to segments *a* and *d*, respectively. In the case of **1231**, the edge-stabilizing interaction directions are assigned to a pair of segments (*a* and *b*) connected at one end of the core, while the strands are linked through a pair of parallel interaction directions assigned to the segments on the opposite side (*c* and *d*).

In summary, the necessary condition for a tecton **B** to create separated ladders can be formulated as follows: these isomers should possess a pair of parallel, oppositely directed interaction directions assigned to segments located on one side of the core or connected to one of its ends (edge-stabilizing directions). Furthermore, the interaction directions assigned to the remaining segments should be either parallel or crossing, and importantly, none of these directions can be parallel to the edge-stabilizing directions.

In the case of forming single networks containing **B**, the results obtained for isomer **1113** indicate that this type of phase separation is directly linked to the specific assignments of three interaction directions in this tecton. Specifically, to observe the densely packed brick-wall phase, a molecule of **B** should have a pair of crossing interaction directions on one side of the core (segments *a* and *d*). Moreover, this tecton must have one interaction direction that is opposite to the direction assigned to the diagonal arm segment (say *c*). Importantly, the interaction direction of the remaining segment (*b*) is irrelevant and can be assigned as **1**, **2**, or **3**. This rule is supported by the results obtained from the simulations of the units **1213** and **1313**, both of which formed brick-wall phases and differ from **1113** by the interaction direction assigned to the second position (*b*, underlined).

Regarding isomers **2131** and **1112** that formed the openwork hexagonal structures, certain similarities in the interaction patterns within these assemblies can be identified, providing the necessary conditions for the formation of network **B** with large pores to be formulated. Both molecules exhibit a pair of uniformly oriented interaction directions: in **1112**, these directions are confined to one side of the molecular core, while in **2131**, they are located on segments connected to one end of the core (underlined). When forming the network, a pair of **2131** (and similarly, **1112**) molecules utilize these interactions to construct the pore rims. The remaining pair of interaction directions, which form a 60-degree angle relative to each other, are subsequently utilized to stabilize the triangular nodal motifs that serve as the vertices of the pores. The combination of these properties enables the formation of spacious void spaces, with their rims defined by pairs of molecules connected in a manner that maximizes the length of the connection.

Based on the results obtained for tecton **2121**, it can be concluded that the occurrence of the two polymorphs shown in the bottom part of Figure 3 is facilitated by parallel interaction directions associated with the diagonal arm segments. In this molecule, diagonal arm segments *a* and *c* have opposite interaction orientations (**2**), while the same applies to segments *b* and *d*, which share a common but distinct orientation (**1**). The assignment of distinct oppositely oriented interactions to pairs of diagonal segments, (*a,c*) and (*b*,*d*), in **B** proves to be a key factor, playing a similar role for the unit **1111** (also centrosymmetric), which is capable of forming the denser Kagome phase. An isomer worth mentioning here is **2222**, which satisfies the above general condition but, unlike **1111** and **2121**, here, mixes randomly with **B** (see Figure 1). This molecule, a prototype of various tetrapod molecular tectons (e.g., carboxylic acids) used in the experiment [29,30,31], has previously been found to form both Kagome and brick-wall phases when adsorbed alone [32]. The primary distinction between **2222** and the segregating units **1111** and **2121** lies in the fact that, in the former, all interaction directions are collinear with their respective arm segments. As this property significantly enhances the tendency of **2222** to mix with **A**, the effect associated with pairs of oppositely oriented interactions in the diagonal segments, which is crucial for **1111** and **2121**, is absent in this particular case.

The right part of Figure 2 presents the heterogeneous coordination curves for the systems discussed in this section. Their shape is significantly different from the results characterizing systems where **A** and **B** formed mixed structures. The most notable feature of these dependencies is that *C_AB_* drops nearly to zero when the temperature falls below approximately 0.2, indicating the complete resolution of adsorbed species. The common peaked shape of these curves reflects the initial formation of small mixed structures, followed by their breakdown and the growth of distinct, pure structures. Note that at maximum, *C_AB_* does not exceed approximately 0.75, indicating very limited coordination of **A** by **B** at the transition temperature in each case. Thus, it can be concluded that the pure molecular assemblies grow effectively as separate entities, yet at elevated temperatures. Further lowering of *T* finalizes this process by sorting out the residual units **A** and **B** for incorporation into the complementary extended structures. The less steep shape of the curves for **1212** and **1132** in the vicinity of the transition temperature (just above it) is associated with the formation of chains and ladders. In these cases, the growth process is more continuous due to the increased stability of multiple chains and ladder fragments, with only the end molecules being more likely to detach. A similar situation was previously observed for isomer **1331**, which formed mixed ladders with **A** (see the left part of Figure 2). A noteworthy effect is the non-zero contact value $C_{AB}\approx 0.16$ predicted for isomer **1322**. This effect can be explained by the residual formation of mixed **A**–**B** chain fragments, as illustrated in the inset of the corresponding panel in Figure 3. Although occurring much less frequently than the pure chains of **B**, the mixed connections create local configurations where each molecule of **B** interacts with two **A** units. As a result, *C_AB_* does not fully diminish to zero even at very low temperatures.

### 2.3. Complex Structure Formation

In the previous sections, we analyzed adsorbed systems where the dominant phase behavior involved the mixing or segregation of components **A** and **B**, resulting in more or less ordered assemblies. In the following, we consider those isomers of **B** for which simple classification was problematic due to the observed formation of both mixed and separated ordered networks, or disordered mixed assemblies with only traces of local ordering.

**Figure 4:**
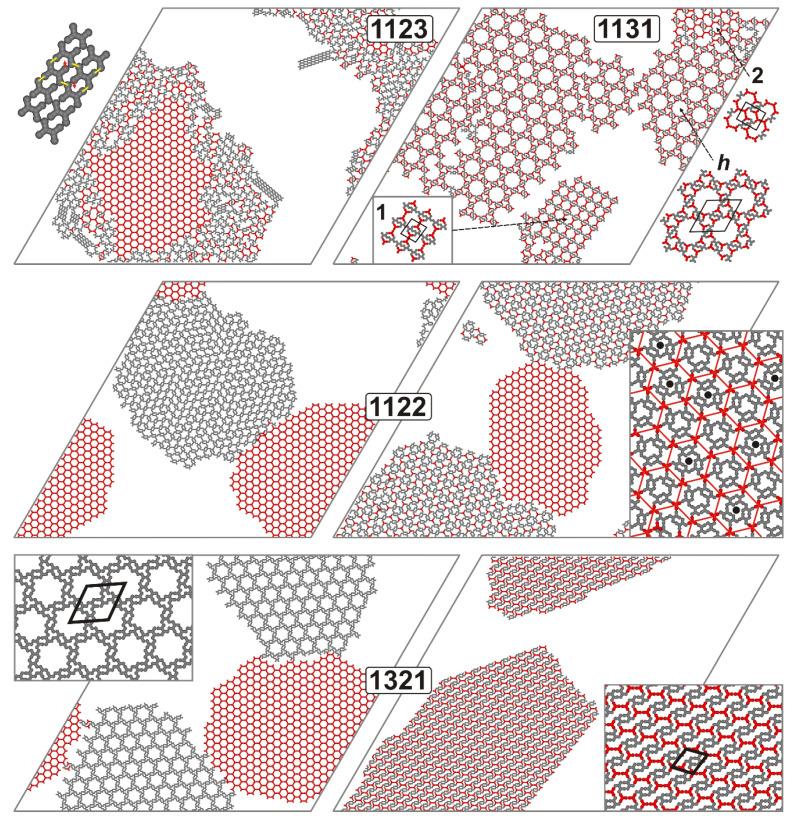
Snapshots of the adsorbed overlayers consisting of 800 molecules of **A** (red) and 800 molecules of **B** (gray), with the latter component exhibiting different sets of interaction directions, as indicated in the respective panels. The thick black lines outline the unit cells of the corresponding ordered networks. A magnified fragment of the ladder formed by **1123**, along with the saturated bonds (yellow) and dangling bonds (red), is shown next to the corresponding snapshot. For the **1131** unit, three network polymorphs—hexagonal (*h*) and brick wall (1 and 2)—are presented. L=200, T=0.01, θ=0.20.

We begin the discussion with the most disordered and multiphase overlayers, represented by isomers **1123** and **1131**, as shown in the top panels of Figure 4. A clear effect observed for the first tecton is the segregation of a pure **A** network within the disordered, **B**-enriched mixture of the adsorbed species. The only indication of **B** ordering in the latter assembly is the formation of sparse ladder-like structures, as shown in the corresponding inset. Importantly, these ladders are not fully energetically optimal structures, as each contributing **B** unit forms only three interactions with its neighbors (indicated by yellow lines) and has one arm segment with an unpaired interaction direction (marked by red arrows). Nevertheless, in these structures, **1123** achieves the maximum possible coordination number of 3, which also characterizes the mixed portion of the assembly.

**Figure 5:**
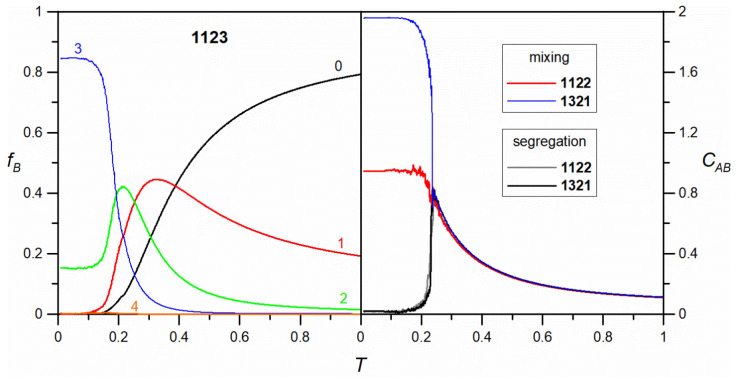
The effect of temperature on the fraction of **B** (**1123**) molecules with a given number of linked molecules (**A** and **B**), *f_B_*, indicated by the numbers 0–4 (left), and on the average number of interactions, *C**_AB_***, formed by a single tripod molecule of **A** with neighboring **B** molecules (right) for the dichotomous self-assembly of **1122** and **1321** leading to component mixing and segregation. L=200, T=0.01, θ=0.20.

To demonstrate this, we calculated the temperature dependence of the fractions of molecules of **B** interacting with 0 to 4 other molecules (both **A** and **B**), as shown in Figure 5. A striking observation is the complete absence of fully coordinated **B** molecules (with four interactions, orange curve) across the entire temperature range. When the temperature drops below approximately 0.25 and goes to zero, molecular condensation results in about 84% of molecules being triply coordinated and around 16% being doubly coordinated. In this final state, due to the specific interaction directions that strongly inhibit full coordination, the **1123** tecton utilizes its three arms in various configurations, leading to the formation of a mixed disordered domain and isolated, fragmented ladders. Similar phase behavior, with even more pronounced ladder formation, was observed for isomer **1223** (see Figures S3 and S4). A possible explanation for the similar effects observed in **1123** and **1223** is the presence of crossing interaction directions on one side of the molecular core (specifically, the last two directions, **2** and **3**), combined with a pair of non-parallel interaction directions on the opposite side.

The top-right section of Figure 4 provides another example of a partially ordered system, where fragments of mixed porous phases were locally formed by **1131**. The insets show magnified views of the porous networks, displaying honeycomb (*h*) and brick-wall-type (1 and 2) topologies, respectively. The first two networks are 1:1 stoichiometric and are characterized by rhombic ($\sqrt{201}$, ∝=60.00°, h) and rectangular ($\sqrt{84}\times \sqrt{63}$, 1) unit cells. The third network, present in much smaller quantities, is a 2:1 (A:B) nonstoichiometric structure with a parallelogram unit cell ($\sqrt{48}\times \sqrt{57}$, ∝=83.41°, 2). The unit cell of the hexagonal network with large pores consists of six molecules of **A** and six molecules of **B**, whereas the unit cells of brick-wall networks 1 and 2 contain two molecules of each type. The mixed networks formed locally by **1131** were observed frequently in the simulations (in different proportions) and reveal the increased tendency of this tecton for the easy formation of polymorphic structures with a 1:1 composition, which agrees with the overall proportions of **A** and **B** in the overlayer. This compositional match is a factor that ensures the equal consumption of molecules as they are incorporated into the growing equimolar networks. However, due to the diverse ways in which **1131** can be fully coordinated and form distinct (and also ordered) phases, these phases become highly competitive, leading to the complex morphology of the adsorbed mixture.

In the next example, **1122** demonstrates a dichotomous self-assembly scenario, resulting in either a pure disordered (aperiodic but continuous) domain of this tecton or a mixed superstructure with distinct periodic features, as illustrated in Figure 4, with nearly equal probability. In the mixed domain, the molecules of **1122** form six-molecular clusters rotated by 60 degrees relative to one another. These differently oriented clusters are interconnected by molecules of **A**, which are periodically arranged to form a hexagonal grid (see the red lines). The orientation of the **1122** clusters embedded in the network of **A** is purely random (left or right), as the manner in which clusters of both types are linked to the network is identical. Consequently, this mixed architecture offers a higher degree of ordering for **1122** (though not fully periodic) compared to its pure segregated domain. In contrast to the previous isomer **1131**, the composition of the mixed network is non-stoichiometric, with a proportion of **A** to **B** equal to 1:3.

The dichotomous manner in which the molecules of **A** and **B** can form adsorbed structures was also observed for isomers **1232** and **1321**. In these instances, however, the self-assembly resulted in fully periodic 2D architectures, both for the segregation and mixing of the components. Figure 4 illustrates this situation for **1321**, while the results simulated for the other tecton are shown in Figure S3 of the Supporting Information section. In the case of segregation, the molecules of **1321** self-assembled into a hexagonal network with spacious pores (43 sites) and a rhombic unit cell ($\sqrt{111}$, ∝ =60.00°) consisting of six molecules of **A** and six molecules of **B**. The mixed, denser structure formed by this isomer is characterized by a parallelogram-shaped unit cell ($\sqrt{39}\times \sqrt{43}$, ∝=68.51°) with an equimolar composition (**2A** + **2B**). A defining feature of this superstructure is the presence of elongated pores, whose edges are formed of four molecules of each component.

The distinct self-assembly mechanisms of the **1122** and **1321** isomers are also evident in the corresponding coordination plots shown in the right panel of Figure 5. In the case of segregation, the curves for both tectons are nearly identical, showing a distinct drop to zero at *T* ≈ 0.25. This effect indicates the initial nucleation of molecules as the temperature decreases from 1 to 0.25, forming irregular connections with **A**, followed by component self-sorting, where **A**–**B** contacts are almost entirely eliminated. Conversely, mixing in the examined systems results in temperature dependencies that differ significantly between **1122** and **1321**. In this case, the initial nucleation of molecules at *T* > 0.25 occurs similarly to segregation, with identical curves. However, at the transition temperature, the average coordination *C**_AB_*** increases, reaching a plateau of approximately 1 for **1122** and 2 for **1321**. The contact value for the latter molecule (*T* → 0) has a more straightforward interpretation, as each **B** molecule in the corresponding mixed domain is surrounded by two neighboring **A** molecules. For **1321**, the non-stoichiometric mixed 1:3 domain contains only one-third of the total number of **A** molecules, while the remaining **A** molecules form a pure honeycomb network. Each **A** molecule in this one-third portion is connected to three **B** units, while the remaining **A** molecules in the pure network do not contribute to *C**_AB_***. As a result, the average heterogeneous coordination of **B** is approximately 1. In the case of isomer **1232**, the obtained temperature dependencies corresponding to segregation and mixing were very similar to those of **1321**, as illustrated in Figure S4.

Table 1 presents a summary of our results, categorizing them into mixed and segregated assemblies, along with more complex self-assembly scenarios. Additionally, Table S1 provides the quantitative parameters of the ordered phases observed in the simulations. The grouping of isomers presented in Table 1 can serve as a guide for the preliminary selection of tetrapod molecules to design 2D adsorbed structures with desired properties. Based on this information, molecular building blocks with various functional centers (atoms or groups) that enable directional interactions can be selected to construct superstructures sustained not only by weak intermolecular forces (e.g., hydrogen bonding) but also by covalent bonds. For example, when the considered model interaction centers are halogen atoms attached to a polyaromatic backbone of **B**, the structures presented in our work can be useful for optimizing on-surface synthetic reactions, such as the Ullmann coupling. In this context, these theoretical predictions could have a potential impact on the design and fabrication of real, persistent, low-dimensional structures composed of mixtures of molecular tectons with different symmetries.

## 3. The Model and Calculations

**Figure 6:**
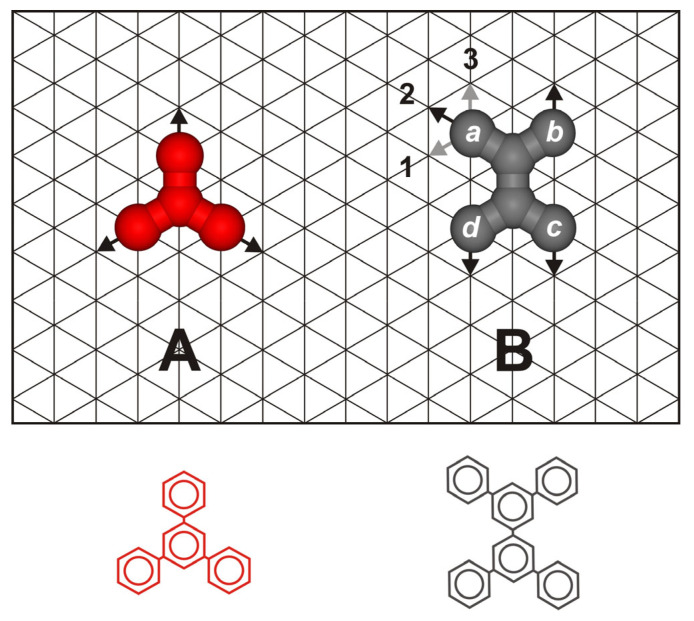
The schematic structure of the tripod (**A**) and tetrapod (**B**) molecules adsorbed on a triangular lattice. The black arrows indicate the interaction directions assigned to each component. For molecule **A**, these directions were fixed, while for molecule **B**, for each arm segment, *a*, *b*, *c*, and *d*, three directions were possible, 1, 2, and 3, corresponding to the *meta* (1,3) and *para* (3) positions in a benzene ring. In the example shown on the right, the active interaction direction in segment *a* is represented by number 2 (indicated by the black arrow). Using the same numbering scheme for the remaining segments of tecton **B** (labeled *b*, *c*, *d*), it was designated as **2131**. The molecular structures illustrated in the bottom image depict possible real polyphenyl tectons corresponding to model tectons **A** and **B**.

The coarse-grained model proposed in this section aimed to predict the self-assembly of a mixture of functional tectons **A** and **B** on a solid (111) crystalline surface under high-vacuum conditions. In this approach, the adsorbing surface was represented by a triangular lattice of equivalent adsorption sites with lattice constant *l*, while the molecules were modeled as collections of discrete segments arranged in tripod (**A**) [23] and tetrapod shapes (**B**) [32]. When the segments are viewed as benzene rings, units **A** and **B** can represent, for example, polyaromatic functional molecules, which have been widely used in 2D crystal engineering. The tripod tecton consisted of three segments, a central core segment and three arm segments, while the tetrapod tecton was composed of six segments: two core segments and four arm segments, *a*, *b*, *c*, and *d*. The inter-segment distance for both components was assumed to be identical and equal to *l*, with each segment occupying a single adsorption site. Figure 6 schematically shows components **A** and **B** adsorbed on a triangular lattice.

Regarding the interaction between units **A** and **B**, we assumed that both species are equipped with identical active centers that facilitate short-range attractive interactions. These centers can represent, but are not limited to, functional groups such as carboxyl or aldehyde, which are commonly used to form supramolecular structures on crystalline surfaces. Since the aforementioned active centers were identical, the corresponding interactions between like pairs (**A**-**A** and **B**-**B**) and unlike pairs (**A**-**B**) were assumed to be the same. Specifically, molecules of **A** were equipped with active arm segments that had fixed interaction directions, oriented along the arms (black arrows). Molecules of **B** possessed active arm segments each with an assigned unique interaction direction, 1, 2, or 3, as illustrated in Figure 6 (see the arrows next to segment *a*). To differentiate these structures, each isomer of **B** was named according to the sequence of interaction directions assigned to the segments *a*, *b*, *c*, and *d*; code *abcd*. In the example shown in Figure 6, the general code *abcd* is equivalent to **2131**. When the segments are assumed to represent benzene rings in the corresponding polyaromatic tectons, the interaction directions 1, 3, and 2 indicated in Figure 6 reflect the *meta* and *para* positions of the attached functional groups (e.g., carboxylic). The interaction between a pair of adsorbed molecules was effective only when their arm segments occupied neighboring lattice sites and the corresponding interaction directions were collinear (→←, see Figure 6). In this case, the energy of the elementary segment–segment interaction was equal to ε. For all other relative positions of a pair of molecules, the interaction energy was zero. Similarly, since the adsorbing surface was assumed to be energetically homogeneous (composed of adsorption sites with identical properties), the segment–surface interaction energy was neglected in the calculations and set to zero for convenience.

The simulations were conducted using the conventional canonical ensemble Monte Carlo method with Metropolis sampling, in which the system size, *L*; temperature, *T*; and number of molecules, *N*, are kept constant. For this purpose, a rhombic fragment of a triangular lattice with side length *L =* 200 (representing adsorption sites) was used, and the initial temperature was fixed. To eliminate finite-size effects, periodic boundary conditions in both planar directions were used. In the first step of the simulation, a mixture of *N_A_* tripod-shaped and *N_B_* tetrapod-shaped molecules was randomly distributed on the lattice. The composition of the adsorbed mixture was characterized by the molar fractions $x_{A}=N_{A}/N$ and $x_{B}=1-x_{A}$, where $N=N_{A}+N_{B}$. The overlayer was equilibrated through a series of Monte Carlo (MC) steps, with each step consisting of a sequence of trial moves carried out as follows. First, a molecule was randomly selected, and its potential interaction energy, *U_o_*, was calculated at its current position. For this purpose, all elementary directional interactions between the selected molecule and its neighboring molecules were considered. These interactions were effective only when collinear (→←), with each contributing an energy of ε = −1. Next, the selected molecule was moved to a new random position on the lattice and randomly rotated in-plane by a multiple of 60 degrees. If there was sufficient free space at the new position to accommodate the molecule, its potential energy, *U_n_*, was calculated using the same procedure as for *U_o_*. Otherwise, the molecule was returned to its original position. To accept or reject the new molecular configuration, the acceptance probability $p=min[1,\exp\left(-\frac{∆U}{kT}\right)]$, where $∆U=U_{n}-U_{o}$ and *k* stands for the Boltzmann constant, was calculated. The obtained value of *p* was compared with a uniformly distributed random number r∈(0,1). The new configuration was accepted when r<p; in the opposite case, the molecule was moved back to its original position. The temperature, *T*, is a crucial variable in our model, as it influences the probability of molecular linkage. At low values of *T*, the likelihood of forming stable intermolecular bonds increases, causing the adsorbed molecules to assemble into superstructures, which, in turn, raises the net potential energy of the system. On the other hand, high temperatures reduce the acceptance probability and lead to the disintegration of existing bonds. These effects are clearly reflected in the shape of the coordination curves *C**_AB_***, which tend to zero at high temperatures and increase rapidly as *T* tends to zero. Similarly, the fraction of **B** molecules with a given number *n* of linked molecules $f_{B}$ is the highest for n=0 (uncoordinated molecules) at high temperatures, while this tendency shifts toward n>0 as the temperature decreases.

During the simulation run, the procedure described above (one MC step) was repeated multiple times, typically $N\times 10^{8}$ times. To determine the quantitative characteristics of the modeled systems, the last ten percent of the total number of MC steps was used for averaging. To minimize the risk of trapping the modeled assemblies in metastable states, the adsorbed overlayer was slowly and linearly cooled from the starting temperature of 1.0 down to the target temperature of 0.01. This was carried out using 1000 decrements, each consisting of 10^5^ MC steps. The simulations were carried out using custom computer codes written in the FORTRAN programming language. For this purpose, an Intel Xeon cluster with a Linux operating system was used, with each modeled system replica running as a separate thread. A single simulation run (one thread) typically lasted over a dozen hours. The energies and temperatures of the model were expressed in units of ε and $\left|\varepsilon \right|/k$, respectively. Surface coverage *θ* was defined as the average number of molecular segments per lattice site, that is, $(4N_{A}+6N_{B})/L^{2}$. In the case of ordered phases, the density ρ was defined as the number of molecular segments per unit cell area. The quantitative functional dependencies presented here are averages over ten independent system replicas. All the results presented in this work were obtained from equimolar mixtures, with $x_{A}=0.5$, consisting of a total of 1600 molecules.

## 4. Conclusions

The results presented in this study demonstrate that the mixed self-assembly of functional units with significantly different symmetries is highly sensitive to the intramolecular distribution of interaction directions within the larger tetrapod molecule. The simplified coarse-grained Monte Carlo (MC) modeling enabled the examination of all 27 tetrasubstituted positional isomers of **B** and their categorization based on the structural type formed during their 1:1 mixed self-assembly with the tripod tecton. While the synthesis of probes and subsequent STM investigation of these candidate **A**/**B** tectons can be tedious in practice, the proposed theoretical approach offers a valuable alternative, reducing the time and resources needed for selecting the optimal tecton. Moreover, the methods employed can be easily extended to other molecular units where short-range interactions play a decisive role in structure formation. This also applies to non-equimolar mixtures, where the composition can be precisely tuned to construct 2D architectures with varying porosity, periodicity, connectivity, and more.

## Figures and Tables

**Table 1:** The classification of **B** isomers based on the structural type formed in an equimolar mixture with **A**. (*) Two polymorphs; (*a*) pure aperiodic and mixed nonstoichiometric (1:3); (*b*) disordered with ladder formation; (*c*) three network polymorphs with 1:1 composition; (*d*) pure and mixed 1:1 domains, both periodic.

Structural Type	Mixed	Segregated
Periodic networks	1221 *, **1222**, **2122**, **2132**	**1111**, **1112**, **1113**, 1121 *, **1213**, **1313**, 2121 *, **2131**
Aperiodic networks	**2222**, **2231**	
Ladders	**1331**	**1132**, **1133**, **1231**
Chains		**1212**, **1322**, **3131**
Complex		
**1122** *^a^*, **1123** *^b^*, **1223** *^b^*, **1131** *^c^*, **1232** *^d^*, **1321** *^d^*		

## Data Availability

Data used in this paper is available to view by contacting the authors.

## Supplementary Materials

The following supporting information can be downloaded at: https://www.mdpi.com/article/10.3390/molecules30040866/s1, Figure S1: Survey of tetrapod tectons with varied assigned interaction directions; Figure S2 and Figure S3: Snapshots of the adsorbed overlayers consisting of 800 molecules of **A** and 800 molecules of **B**, with the latter component exhibiting different sets of interaction directions; Figure S4: Effect of temperature on the fraction of **B** (1223) molecules with a given number of linked molecules, $\;f_{B}$ and on the average number of interactions, *C**_AB_***; Table S1: Parameters of adsorbed ordered structures consisting of pure **B** and its mixture with **A**.
