# Supplementary material for: Theoretical Modeling of the Structure Formation in Adsorbed Overlayers Comprising Molecular Building Blocks with Different Symmetries
Source: Molecules. 2025 Feb 13;30(4):866. doi: 10.3390/molecules30040866 (PMC11858382; doi:10.3390/molecules30040866)
Supplement: Supplementary file 1 [file molecules-30-00866-s001.zip › molecules-3466574-supplementary.pdf]

Supporting Information

for

Theoretical Modeling of the Structure Formation in Adsorbed Overlayers Comprising Molecular Building Blocks with Different Symmetries

Paweł Szabelski*

Department of Theoretical Chemistry, Institute of Chemical Sciences, Faculty of Chemistry, Maria Curie-Skłodowska University, Pl. M.C. Skłodowskiej 3, 20-031 Lublin, Poland

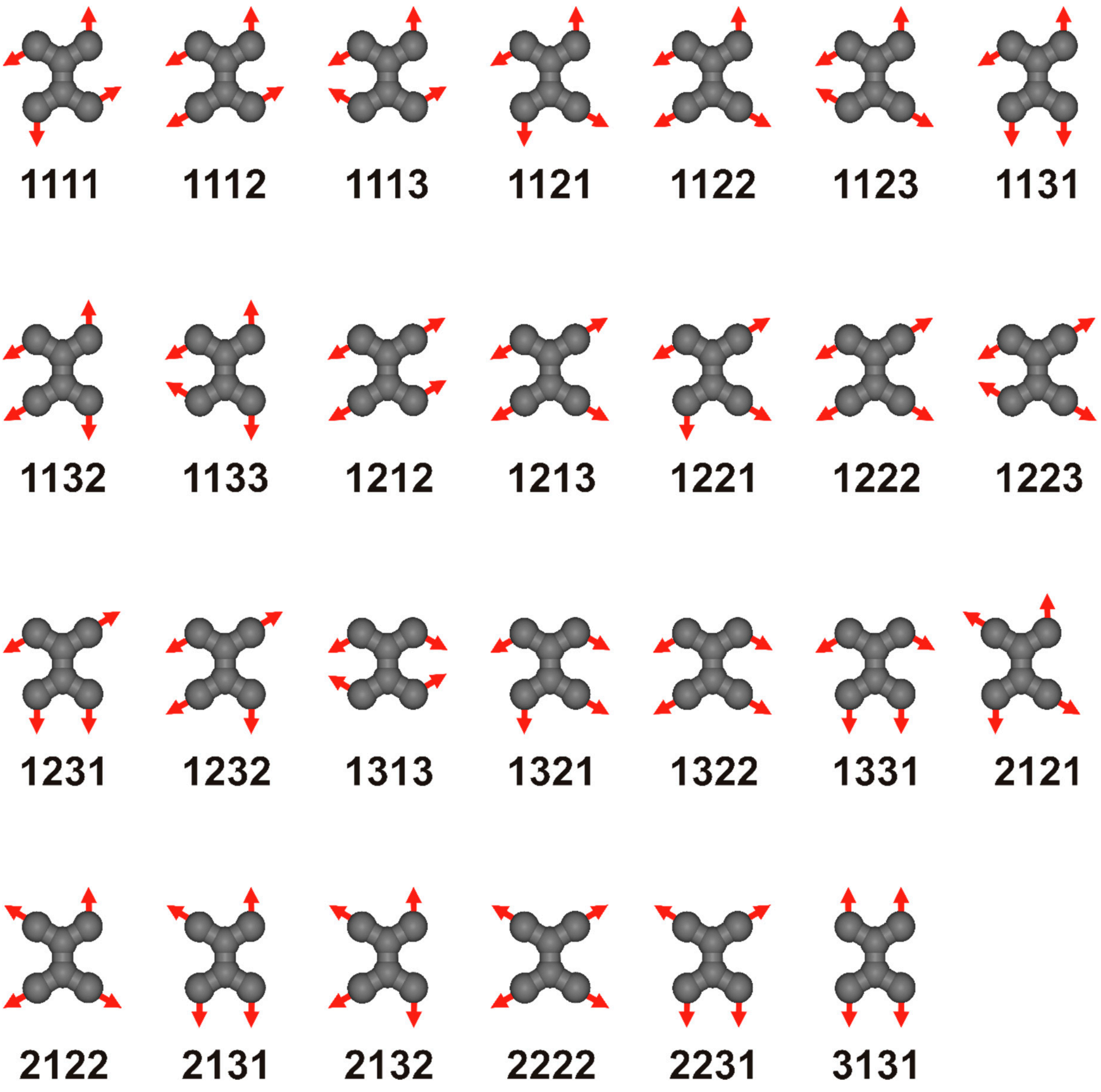

Figure S1. Survey of tetrapod tectons with varied assigned interaction directions (red arrows).

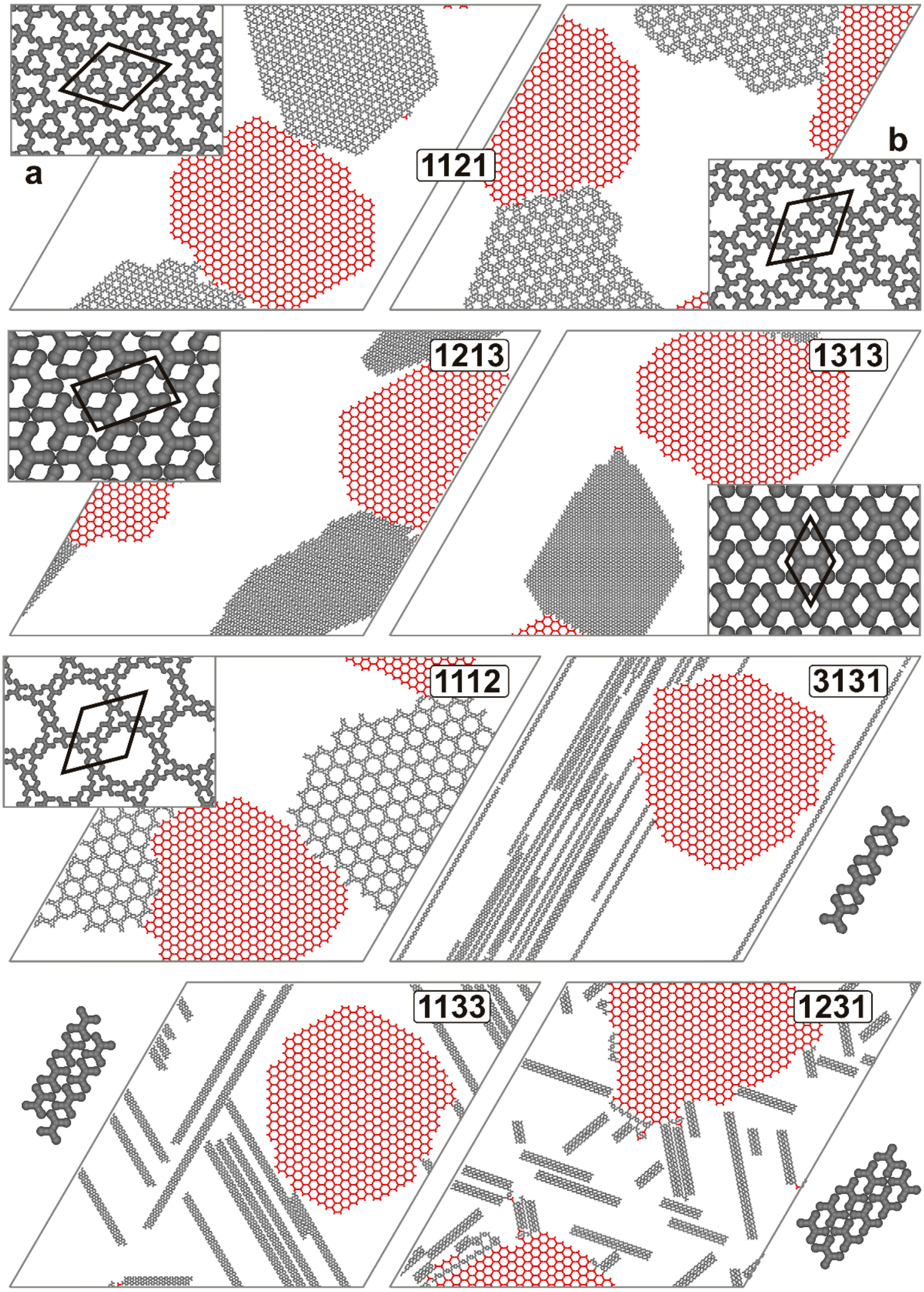

Figure S2. Snapshots of the adsorbed overlayers consisting of 800 molecules of **A** (red) and 800 molecules of **B** (grey), with the latter component exhibiting different sets of interaction directions as indicated in the respective panels. The thick black lines outline the unit cells of the corresponding ordered networks, including polymorphs **a** and **b** comprising **1121**. Magnified fragments of the chains and ladders formed by **3131**, **1133** and **1231** are displayed next to the corresponding snapshots. $L = 200$, $T = 0.01$, $\theta = 0.20$.

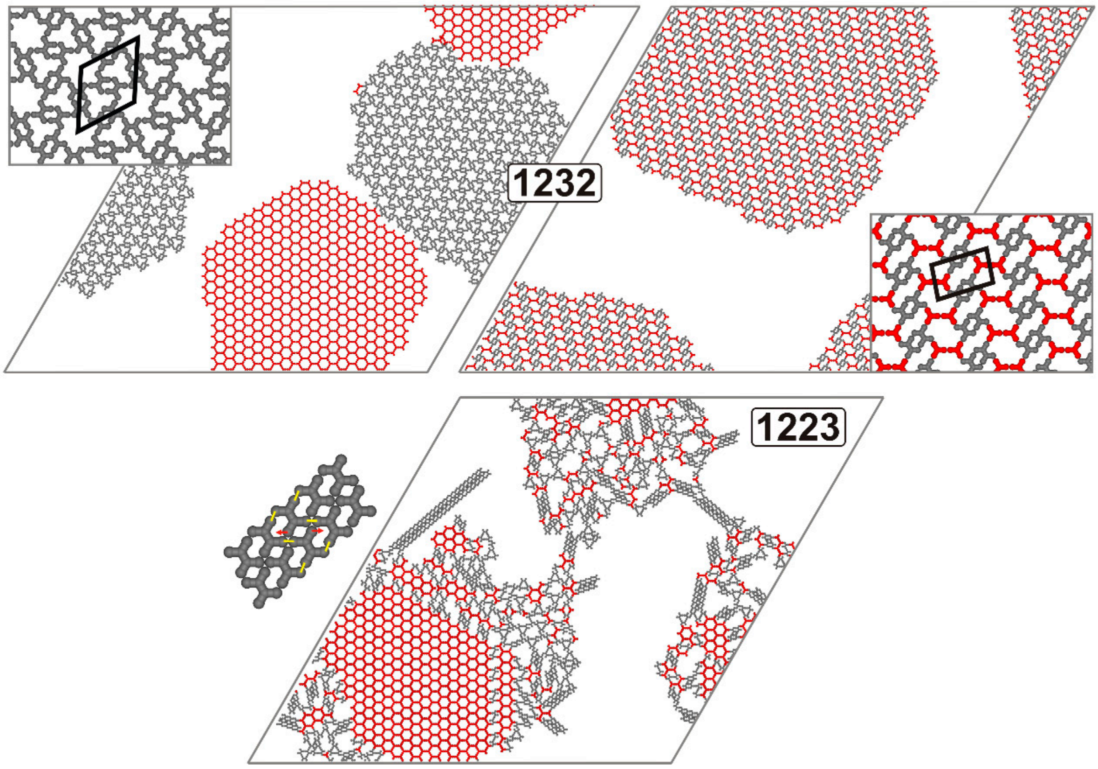

Figure S3. Snapshots of the adsorbed overlayers consisting of 800 molecules of **A** (red) and 800 molecules of **B** (grey), with the latter component exhibiting different sets of interaction directions as indicated in the respective panels. The thick black lines outline the unit cells of the corresponding ordered networks. A magnified fragment of the ladder formed by **1223**, along with the saturated bonds (yellow) and dangling bonds (red) bonds is shown next to the corresponding snapshot. $L = 200$, $T = 0.01$, $\theta = 0.20$.

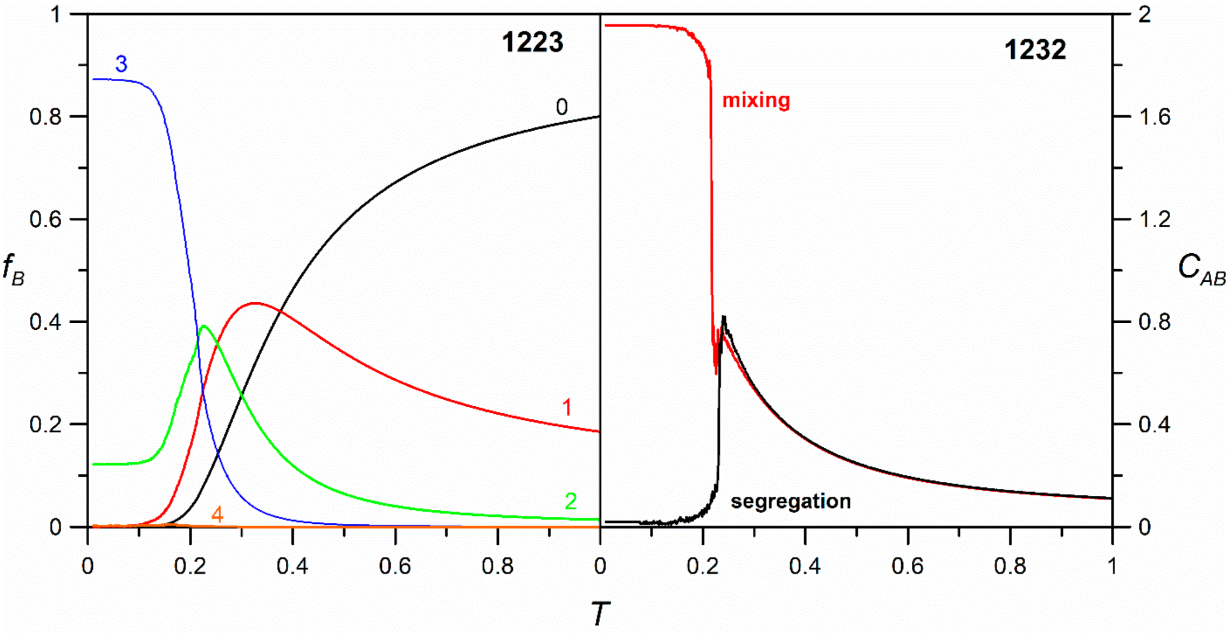

Figure S4. Effect of temperature on the fraction of **B** (**1223**) molecules with a given number of linked molecules (**A** and **B**), f_B, indicated by the numbers 0-4 (left), and on the average number of interactions, C_{AB}, formed by a single tripod molecule **A** with neighboring **B** molecules (right) - for the dichotomous self-assembly of **1232** leading to component mixing and segregation. $L = 200$, $T = 0.01$, $\theta = 0.20$.

molecule/ polymorph	structure type	unit cell parameters	no. of molecules per unit cell	density ρ
**1213**	closely packed	parallelogram $3 \times \sqrt{31}$, $\propto= 68.95°$	2	0.770
**1313**	closely packed	rhombic 3×3, $\propto= 60.00°$	1	0.770
**1112**	openwork honeycomb	rhombic $3\sqrt{13} \times 3\sqrt{13}$, $\propto= 60.00°$	6	0.355
**1113**	dense brickwall	parallelogram $3 \times \sqrt{57}$, $\propto= 53.41°$	2	0.660
**1111**	Kagome	rhombic $\sqrt{39} \times \sqrt{39}$, $\propto= 60.00°$	3	0.533
**2121*b***	brickwall	parallelogram $2\sqrt{3} \times \sqrt{13}$, $\propto= 76.10°$	1	0.495
**2121*k***	Kagome	rhombic 7×7, $\propto= 60.00°$	3	0.424
**2131**	openwork honeycomb	rhombic $2\sqrt{37} \times 2\sqrt{37}$, $\propto= 60.00°$	6	0.281
**1121_a**	hexagonal porous	rhombic $\sqrt{79} \times \sqrt{79}$, $\propto= 60.00°$	6	0.526
**1121_b**	hexagonal porous	rhombic $2\sqrt{21} \times 2\sqrt{21}$, $\propto= 60.00°$	6	0.495
**1321**	openwork honeycomb	rhombic $\sqrt{111} \times \sqrt{111}$, $\propto = 60.00°$	6	0.374
**1221_1**	porous 1:1	parallelogram $\sqrt{31} \times \sqrt{67}$, $\propto= 63.27°$	2A+2B	0.491
**1221_2**	porous 1:1	parallelogram $\sqrt{37} \times 7$, $\propto= 63.50°$	2A+2B	0.525
**2122**	porous 1:1	parallelogram $\sqrt{37} \times \sqrt{67}$, $\propto= 73.07°$	2A+2B	0.420
**2132**	porous 1:1	parallelogram $\sqrt{37} \times \sqrt{57}$, $\propto= 78.70°$	2A+2B	0.444
**1222**	porous 1:1	parallelogram $7 \times \sqrt{61}$, $\propto= 86.33°$	2A+2B	0.366
**1232**	porous 1:1	parallelogram $2\sqrt{7} \times \sqrt{79}$, $\propto= 83.90°$	2A+2B	0.428
**1321**	porous 1:1	parallelogram $\sqrt{39} \times \sqrt{43}$, $\propto= 68.51°$	2A+2B	0.525
**1131_1**	porous brickwall 1:1	parallelogram $4\sqrt{3} \times \sqrt{57}$, $\propto= 83.41°$	2A+2B	0.385
**1131_2**	porous 2:1	rectangular $2\sqrt{21} \times 3\sqrt{7}$, $\propto= 90°$	4A+2B	0.385
**1131_h**	porous honeycomb 1:1	rhombic $\sqrt{201} \times \sqrt{201}$, $\propto = 60.00°$	6A+6B	0.344
**1122**	1:3, partially periodic (A)	A: rhombic $3\sqrt{13} \times 3\sqrt{13}$, $\propto = 60.00°$	2A+6B	0.434

Table S1. Parameters of adsorbed ordered structures consisting of pure **B** (segregation, highlighted in pale red) and its mixture with **A** (mixing, highlighted in gray).
